# Multiple Anesthesia/Surgery Cannot Impair Reference Memory in Adult Mice

**DOI:** 10.1155/2020/3736912

**Published:** 2020-03-07

**Authors:** Xiaoxin Zhou, Jian Lu, Tong Wu, Xuliang Jiang, Weitian Tian, Wanbing Dai, Siyi Qi, Xuemei Chen, Jiaqiang Zhang, Diansan Su

**Affiliations:** ^1^Department of Anesthesiology, Renji Hospital, Shanghai Jiaotong University, School of Medicine, Shanghai, China; ^2^Department of Anesthesiology, The Second Affiliated Hospital of Jiaxing University, Jiaxing City, Zhejiang Province, China; ^3^Department of Anesthesiology and Perioperative Medicine, Henan Provincial People's Hospital, People's Hospital of Zhengzhou University, Zhengzhou, Henan Province, China

## Abstract

Postoperative cognitive dysfunction increases mortality and morbidity in perioperative patients. Numerous studies have demonstrated that multiple surgery/anesthesia during the neurodevelopmental period affects cognitive function, whereas a single anesthesia/surgery rarely causes cognitive dysfunction in adults. However, whether adults who undergo multiple anesthesia/surgery over a short period will experience cognitive dysfunction remains unclear. In this study, central nervous system inflammation and changes in cholinergic markers were investigated in adult mice subjected to multiple laparotomy procedures over a short period of time. The results showed that despite the increased expression of IL-6 and TNF-*α* in the hippocampus after multiple operations and the activation of microglia, multiple anesthesia/surgery did not cause a decline in cognitive function in adult mice. There were no changes in the cholinergic markers after multiple anesthesia/surgery.

## 1. Introduction

Postoperative cognitive dysfunction (POCD) is a common complication after a major surgery [1–3]. The condition is characterized by impaired learning and memory and may persist for months and years after surgery [4, 5]. POCD is thought to be associated with major surgery and advanced age [6, 7], and there is no doubt that older individuals are more likely to develop POCD [8]. Although POCD does occur in adults in clinical settings, whether adult mice develop similar learning and memory impairment after anesthesia/surgery remains controversial.

Lin and Zuo demonstrated that 4-month-old male rats exposed to isoflurane had significant impairments in long-term spatial memory assessed using a Barnes maze in addition to impaired hippocampus-dependent learning and memory in a fear conditioning test [9]. However, our previous work showed that single anesthesia/surgery induced memory decline and attenuated central cholinergic biomarkers in aged mice but not in adult mice [10]. Walters et al. [11] demonstrated that exposure to anesthesia alone does not cause persistent learning and memory impairments in adult monkeys. Nearly all studies on adult animals have focused on single anesthesia/surgery, and few have explored what happens to adult mice after multiple anesthesia/surgery. In the present study, we hypothesized that multiple anesthesia/surgery would impair the reference memory in adult mice.

## 2. Materials and Methods

Ethical approval for this study was provided by the Animal Care and Use Committee of Shanghai Jiao Tong University, School of Medicine. All animal procedures were performed in accordance with the National Institutes of Health animal care guidelines.

### 2.1. Animals and Anesthesia/Surgery Procedure

C57BL/6J mice (aged 8 weeks, male) were purchased from the Animal Research Center of Shanghai Jiaotong University, School of Medicine. The animals were housed in standard cages (size, 325 × 210 × 180 mm, 4-5 mice per cage) under controlled laboratory conditions (temperature of 22 ± 2°C, 12 h light/12 h dark cycle) with free access to regular rodent pellets and water. All mice were allowed to adapt to their new environment for 7 days before beginning the experiments.

**Figure 1:**
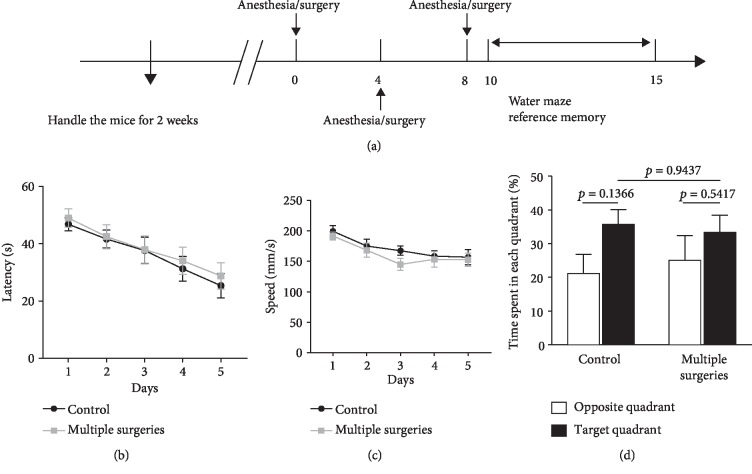
Multiple anesthesia/surgery did not impair spatial reference memory in adult mice. (a) Experimental timeline of surgery procedure and Morris water maze test. Mice in the multiple anesthesia/surgery group underwent surgery every 4 days. After the anesthesia/surgical procedure, training was conducted for 5 days followed by probe tests on day 14. (b) Escape latency to reach the hidden platform during the 5-day training; there was no significant difference between the control group and the multiple surgery group (*p* = 0.9949). (c) Average swimming speed during hidden platform training; there was no significant difference between the control group and the multiple surgery group (*p* = 0.8907). (d) Time spent in the target quadrant during the probe test; there was no significant difference between the control group and the multiple surgery group (*n* = 10 for both control and multiple anesthesia/surgery groups). mA/S: multiple anesthesia/surgery.

The mice were randomly divided into three groups: control group, single anesthesia/surgery group, and multiple anesthesia/surgery group. Exploratory laparotomy was performed under isoflurane anesthesia (induced with 4.0% isoflurane and maintained with 2.0% isoflurane in 0.30 FiO_2_). The mice were gently restrained to a heating pad (37°C) using paper tape, and the whole procedure lasted 10 min. Mice in the single anesthesia/surgery group received surgery once, and those in the multiple anesthesia/surgery group underwent surgery every four days (3 total operations) (Figure 1(a)).

Spatial reference memory in mice from the control and the multiple anesthesia/surgery groups was evaluated in the Morris water maze (MWM) since the second day after the last surgery. In different cohorts of animals, 8 mice from control, single anesthesia/surgery, and multiple anesthesia/surgery groups were euthanized at each time point (6, 24, and 48 h after surgery) in each group. Hippocampus tissue and serum were harvested for the measurements of IL-1*β*, IL-6, TNF-*α*, and IL-10 by enzyme-linked immunosorbent assay (ELISA). Hippocampus tissue was dissected for detecting cholinergic markers using western blot analysis, and the whole brain was dissected for microglial immunofluorescence staining.

### 2.2. Morris Water Maze (MWM)

The MWM test was performed using a computerized video tracking system as previously described [12], and the method description partly reproduces their wording. For the reference memory test, animals were maintained in the same rearing conditions throughout the procedure. The test was performed by an operator blinded to the group conditions. Briefly, a hidden round platform was placed 1 cm below the water surface in the center of the northeast quadrant of a circular pool (110 cm in diameter and 30 cm in depth). The water was maintained at 23–25°C, and the pool was situated in a room with visual cues. The position of the cues remained unchanged throughout the task.

Before the anesthesia/surgical procedure, mice were individually handled for 2 min each day for 1 week. After the anesthesia/surgical procedure, training was conducted for 5 days for the reference memory test (Figure 1(a)). The platform was located in the center of the fourth quadrant. In all trials, the mouse was released into the water facing the pool wall from one of four separate quadrants and allowed to swim until it climbed on to the platform. Once the mouse found the platform, the trial was terminated, and the mouse was allowed to stay on the platform for 15 s. If the mouse failed to find the platform within 60 s, it was gently guided to the platform and allowed to remain on the platform for 15 s. Four trials were conducted every day, separated by a 30–40 min intertrial interval; the platform remained at the same location throughout the test. The amount of time the mouse took to find and mount the platform (escape latency) and the swimming speed were calculated from recorded videos using the MWM software (Shanghai Jiliang Software Technology Co. Ltd., China). The probe test was performed on the fifth day of the reference memory test. In this test, the platform was absent, and the animals were free to swim for 60 s, starting from the quadrant opposite the platform. The times spent in the target and opposite quadrants were recorded.

### 2.3. Enzyme-Linked Immunosorbent Assay (ELISA) for IL-1*β*, IL-6, TNF-*α*, and IL-10

Serum and the hippocampus tissues were collected at indicated time points after surgery in mice from the control, single anesthesia/surgery, and multiple anesthesia/surgery groups. The harvested brain tissues were homogenized on ice using RIPA lysis buffer (Beyotime Biotechnology, Shanghai, China) with protease inhibitors (A32953, Thermo). After collection of the whole blood, it was left undisturbed at room temperature for approximately 30 min. Following centrifugation at 3000 ×*g* for 10 min at 4°C, the supernatant (serum) was collected. Levels of IL-1*β*, TNF-*α*, IL-6, and IL-10 in both serum and hippocampus tissue were measured using mouse ELISA kits (MultiSciences Biotechnology, Hangzhou, China).

### 2.4. Immunofluorescence Staining

The mice were euthanized and subjected to cardiac perfusion with 20 mL precooled saline and then with 20 mL 4% paraformaldehyde. The mouse brain was fixed and dehydrated with 20% and 30% sucrose solution in turn. The mouse brain was subsequently sectioned into 22 *μ*m slices with a freezing microtome (CM3050S, Leica). The brain slices were washed with 0.3% Triton X-100 in PBS and blocked with blocking buffer (Beyotime Biotechnology, Shanghai, China) for 1 h at room temperature. The slices were incubated with primary antibody Iba1 (1 : 100, Abcam) at 4°C overnight. The slices were washed with 0.3% PBST for 30 min and secondary antibody (1 : 1000, Abcam) for 1 h at room temperature. Fluorescence signals were captured using confocal microscopy (FV3000, Olympus).

### 2.5. Western Blotting

Brain tissues were harvested, and protein was extracted as previously described [13]. Protein concentration was quantified using bicinchoninic acid [14] (BCA) assay following the manufacturer's instructions (23225, Thermo). Protein can react with alkaline Cu2^+^ producing cuprous ion (Cu1^+^) which can form an intense purple complex with bicinchoninic acid in an alkaline environment; the basis of BCA is that the color produced from this reaction increases over a broad range of increasing protein concentrations. Thirty micrograms of protein were separated with 8% sodium dodecyl sulfate-polyacrylamide gel electrophoresis and transferred to PVDF membranes (Millipore). Nonspecific binding sites were blocked with 1% bovine serum albumin for 1 h at room temperature and incubated with choline acetyltransferase (ChAT, 1 : 1000; Abcam), acetylcholinesterase (AChE, 1 : 1000; Abcam), and choline transporter (ChT, 1 : 1000; Abcam) at 4°C overnight. The membranes were washed with 0.1% TBST for 30 min and incubated with secondary antibody for 1 h at room temperature. The membranes were detected using an enhanced chemiluminescence system (Yeasen, China) and analyzed using Image Lab 3.0 (Bio-Rad).

### 2.6. Statistical Analysis

Statistical analyses were performed with GraphPad Prism 6.0 (GraphPad Software Inc., USA). Data are presented as the mean ± SEM. A two-way repeated-measures ANOVA was used to analyze the water maze escape latency and average speed. A one-way ANOVA or unpaired *t* test was used to analyze the probe quadrant trial data, probe test data, abundance of cytokines, relative fluorescence quantification, and relative protein levels of ChAT, AChE, and ChT. Statistical significance was determined if *p* < 0.05.

## 3. Results

### 3.1. Multiple Anesthesia/Surgery Did Not Impair Spatial Reference Memory in Adult Mice

The MWM was used to evaluate learning and memory after three incidences of anesthesia/surgery. After the anesthesia/surgical procedure, training was conducted for 5 days, and probe tests were conducted on day 15 (Figure 1(a)). During the training course, the mice from the multiple surgery group did not show longer escape latency compared with the untreated control mice (Figure 1(b)). There was no significant difference in swimming speed between the two groups during the training (Figure 1(c)). During the probe test, the preference for the target quadrant and the time spent looking for the hidden platform were comparable between the control mice and those in the multiple surgery group (Figure 1(d)). These results suggested that multiple anesthesia/surgery does not impair reference memory in young adult mice.

### 3.2. Single and Multiple Anesthesia/Surgery Induced Transient Neuroinflammation in Different Pattern in Adult Mice

**Figure 2:**
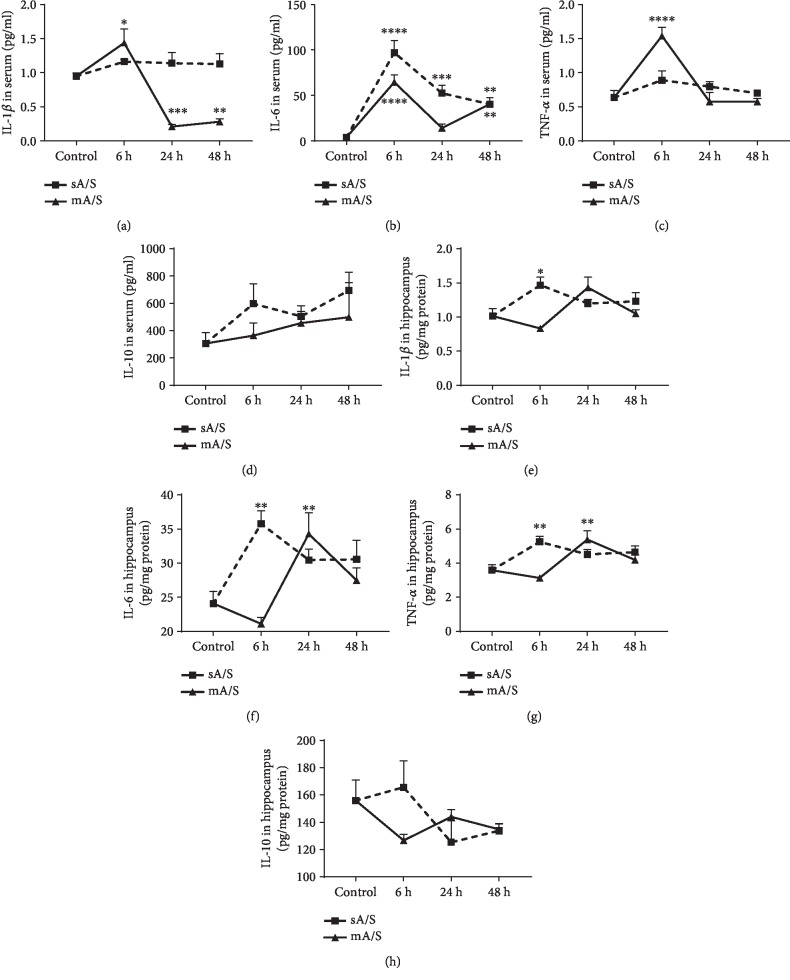
Multiple anesthesia/surgery induced transient central inflammation in hippocampus. (a–c) The levels of IL-1*β*, IL-6, and TNF-*α* increased in blood at 6 h after multiple surgeries (^∗^*p* = 0.0456 for IL-1*β*, ^∗∗∗∗^*p* = 0.0001 for IL-6, and ^∗∗∗∗^*p* < 0.0001 for TNF-*α*). Only IL-6 increased in blood after single surgery (^∗∗∗∗^*p* = 0.0001 at 6 h, ^∗∗∗^*p* = 0.003 at 24 h, and ^∗∗^*p* = 0.0096 at 48 h). IL-1*β* decreased in blood at 24 h and 48 h after multiple surgeries (^∗∗∗^*p* = 0.0007 at 24 h and ^∗∗^*p* = 0.0017 at 48 h). (e–g) IL-6 and TNF-*α* increased in the hippocampus at 24 h after multiple anesthesia/surgery (^∗∗^*p* = 0.0138 for IL-6 and ^∗∗^*p* = 0.0050 for TNF-*α*). IL-1*β*, IL-6, and TNF-*α* increased in the hippocampus at 6 h after single surgery (^∗^*p* = 0.0108 for IL-1*β*, ^∗∗^*p* = 0.001 for IL-6, and ^∗∗^*p* = 0.0035 for TNF-*α*). (d, h) IL-10 in blood and hippocampus had no significant changes after single/multiple surgeries. Mean (SEM), one-way ANOVA. sA/S: single anesthesia/surgery; mA/S: multiple anesthesia/surgery.

Single anesthesia/surgery induced inflammation in both serum and hippocampus at an early stage (6 h after anesthesia/surgery). As shown in Figure 2, IL-6 in serum increased significantly 6 h after single anesthesia/surgery and slightly decreased 24 hours later. The level of IL-1*β*, IL-6, and TNF-*α* in hippocampus increased significantly 6 h after anesthesia/surgery and reduced to a level comparable to the control group at 24 and 48 h after surgery.

Multiple anesthesia/surgery induced an early inflammation in serum, but the inflammatory response in the hippocampus occurred relatively late. As shown in Figure 2, IL-1*β*, IL-6, and TNF-*α* in the serum increased significantly at 6 h after anesthesia/surgery and then decreased 24 hours later. While IL-6 and TNF-*α* in hippocampus increased at 24 h after anesthesia/surgery and decreased at 48 h, both single and multiple anesthesia/surgery did not affect the IL-10 level in serum and hippocampus (Figures 2(d) and 2(h)).

### 3.3. Both Single and Multiple Anesthesia/Surgery Induced Activation of Microglia in the Hippocampus

**Figure 3:**
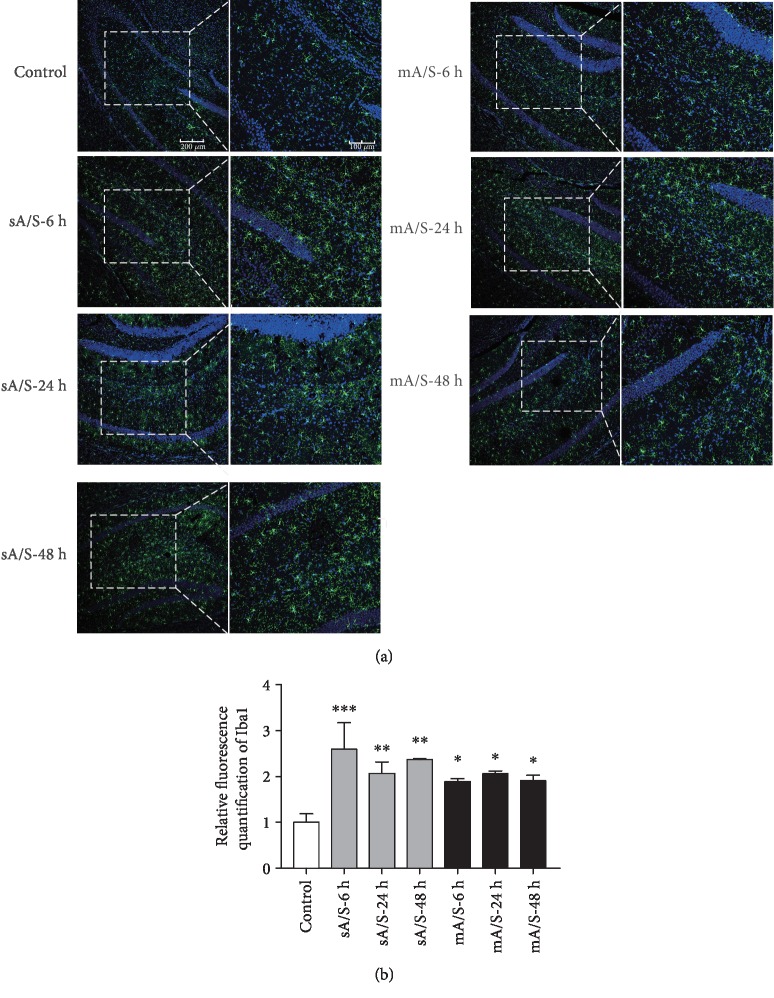
Both single and multiple anesthesia/surgery induced the activation of microglia in the hippocampus. (a) Representative photomicrographs of immunofluorescence of microglia (Iba1, green); nucleus was stained with DAPI (blue). (*n* = 3–5). Scale bar: 200 *μ*M (left) and 100 *μ*M (right). (b) Fluorescence signal intensity was quantified by ImageJ (versus control, *p* = 0.0008 for sA/S-6 h, *p* = 0.0139 for sA/S-24 h, *p* = 0.0034 for sA/S-48 h, *p* = 0.0459 for mA/S-6 h, *p* = 0.0248 for mA/S-24 h, and *p* = 0.0409 for mA/S-48 h). Mean (SEM), one-way ANOVA. sA/S: single anesthesia/surgery; mA/S: multiple anesthesia/surgery.

The activation of microglia is another reflection of central inflammation. We stained the postoperative hippocampal section for Iba1, a marker for microglia, at 6, 24, and 48 h after surgery. The activation of microglia was observed in the single anesthesia/surgery group at all three time points (Figures 3(a) and 3(b)).

### 3.4. Multiple Anesthesia/Surgery Did Not Impair the Central Cholinergic System

**Figure 4:**
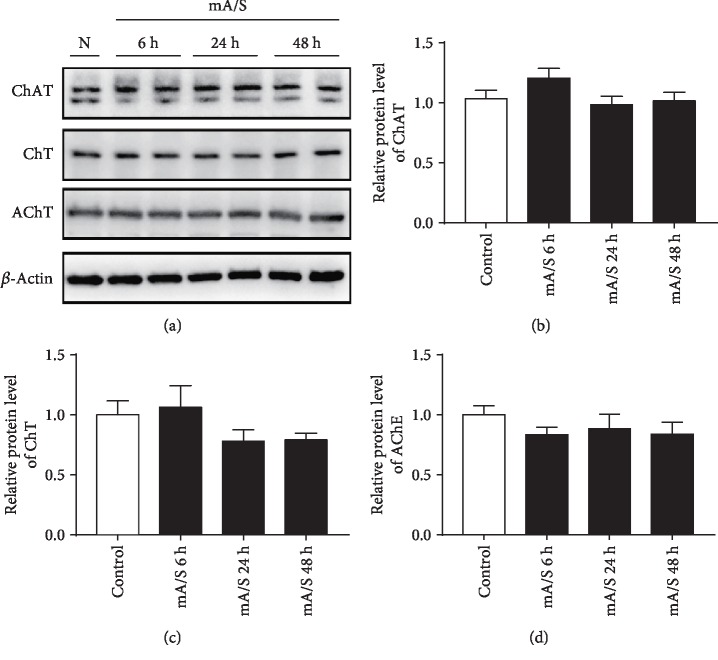
Both single and multiple anesthesia/surgery did not impair the central cholinergic system. (a–d) Representative western blot of ChAT/AChE/ChT proteins and their relative levels in the hippocampus at 6, 24, and 48 hours after surgery. *β*-Actin was used for normalizing protein levels. *n* = 4 for each group. sA/S: single anesthesia/surgery; mA/S: multiple anesthesia/surgery.

We have previously demonstrated that central cholinergic neuronal degeneration promotes the development of POCD in aged mice [10]. Does multiple anesthesia/surgery affect the central cholinergic system? The levels of hippocampal ChAT, AChE, and ChT were measured by western blotting at three time points (6, 24, and 48 h) after surgery. No significant change was shown in the central cholinergic system after multiple operations (Figures 4(a)–4(d)).

## 4. Discussion

In the present study, we investigated whether multiple anesthesia/surgery could impair the spatial reference memory of young adult mice and evaluated the changes in neuroinflammation and the degeneration of central cholinergic neurons after multiple anesthesia/surgery. The results showed that multiple anesthesia/surgery did not impair the spatial reference memory in young adult mice, though the activation of microglia and the increase of proinflammatory cytokines were observed.

Multiple anesthesia/surgery has different effects on the CNS at different developmental stages. Several studies have demonstrated that exposure to multiple anesthesia is neurotoxic to the developing brain [15–18]. However, there has been no study focusing on the effect of multiple anesthesia/surgery on the adult brain. The results from the present study demonstrated that neuroinflammation induced by multiple anesthesia/surgery is not enough to impair the learning and memory of adult mice.

There are several possible reasons why multiple anesthesia/surgery did not induce the impairment of spatial reference memory in adult mice. (A) The immunological system in adult mice is powerful enough to resolve the neuroinflammation in a very short time. Our results showed that the levels of IL-1*β*, IL-6, and TNF-*α* in the hippocampus elevated at 24 h after anesthesia/surgery and all returned to a normal range within 48 h. (B) Neuroinflammation was not severe enough after multiple anesthesia/surgery to cause cognitive decline. (C) The cholinergic neuron is strong enough to undergo multiple anesthesia/surgery because no cholinergic markers in the hippocampus were changed despite the repeated damage.

Accumulating evidence shows that neuroinflammation plays a key role in the development of POCD [19, 20]. The extent of the elevation of proinflammatory cytokines (IL-1*β*, IL-6, TNF-*α*, etc.) in both the central nervous system and the systemic circulation after surgery may relate to the degree of cognitive decline [21, 22]. These proinflammatory cytokines can be transmitted through the impaired blood–brain barrier (BBB) to overactivate microglia, resulting in hippocampal neuroinflammation in POCD [23]. The neural system of aged individuals is more susceptible than that of young adults [24, 25]. Our previous study showed that a single surgery (splenectomy) did not impair the spatial reference memory of young adult mice in spite of the increased IL-1*β*/IL-6 and activated microglia [26]. The powerful immune system may be helpful in reducing and resolving neuroinflammation after anesthesia/surgery. In addition, Shin et al. demonstrated that the BBB was damaged in aged mice, but not adult mice after anesthesia/surgery [27].

Immune tolerance might occur after multiple anesthesia/surgery. Wendeln et al. [28] demonstrated that multiple peripheral inflammatory stimuli induced immune tolerance in the brain, which mainly manifested in the elevation of IL-10 in the brain. However, multiple anesthesia/surgery did not induce the elevation of the IL-10 level. Therefore, whether multiple anesthesia/surgery induces immune tolerance in the brain remains unclear. Liu and his colleagues found that neuroinflammation induced by peripheral inflammation can be mitigated by the precondition of 3 consecutive intraperitoneal injections of *E.coli*; the levels of IL-1*β*, IL-6, and TNF-*α* in the brain were significantly lower than the controlled group when encountered an acute immune challenge [29]. In the present study, we observed a mild decrease (no statistical differences) in the levels of IL-1*β*, IL-6, and TNF-*α* at 6 h in the hippocampus. Suppose the previous two surgeries were the precondition and the last one surgery was an acute immune challenge, the mild decrease of IL-1*β*, IL-6, and TNF-*α* at 6 h might result from such preconditioning which activates anti-inflammatory pathway. But the precondition did not affect the activation of microglia, which might explain the delayed increase of IL-6 and TNF-*α* (secreted by activated microglia) [30, 31].

Maintaining the central cholinergic system might be another reason why multiple anesthesia/surgery has no effects on spatial reference memory. The central cholinergic system is essential for learning and memory, including the modulation of the acquisition, encoding, consolidation, reconsolidation, extinction, and retrieval of memory [32]. Our previous work showed that central cholinergic neuronal degeneration facilitates the development of POCD [10, 33]. In this study, the central cholinergic system was stable after multiple anesthesia/surgery, corresponding to normal learning and memory.

There are some limitations in this study. We only measured the spatial reference memory, and we observed no changes after multiple anesthesia/surgery. We did not investigate how other domains of learning and memory such as working memory changed after multiple anesthesia/surgery. In our previous study, we demonstrated that although the spatial reference memory was not changed after a single anesthesia/surgery, the working memory was impaired in young adult mice [33]. Although exposure to multiple anesthesia/surgery in such a short period is not common in clinical practice, our data from the present study indicated that the effects of multiple anesthesia/surgery on learning and memory are no worse than those of a single anesthesia/surgery.

In conclusion, in spite of mild neuroinflammation, multiple anesthesia/surgery does not impair spatial reference memory in young adult mice. In clinical settings, patients may require multiple anesthesia/surgery in many situations such as anaplasty after burning or orthopedic deformity correction. The results of the present study indicated that multiple anesthesia/surgery might be not a risk factor for learning and memory impairment after surgery in young adult patients.

## Data Availability

The data used to support the findings of this study are included within the article.
